# HIV-specific and resistant CAR T cells promote control of HIV replication in people with HIV

**DOI:** 10.21203/rs.3.rs-10181812/v1

**Published:** 2026-07-03

**Authors:** James Riley, Pablo Tebas, Julie Jadlowsky, Yuepeng Zhang, M. Betina Pampena, Jake Robinson, Lucia Baquero, Andrea Brennan, Irina Kulikovskaya, Vanessa Gonzalez, Amanda Kotch, Max Eldabbas, Chungdhak Tsang, Ola Mohamed, Gregory Laird, Honghong Sun, Zhenyu Huang, Eline Luning Prak, Gabriela Plesa, Katherine Bar, Joseph Fraietta, Donald Siegel, Elizabeth Hexner, James Hoxie, Mary Putt, Michael Betts, Xu Yu, Mathias Lichterfeld

**Affiliations:** University of Pennsylvania; University of Pennsylvania Hospital; University of Pennsylvania; Ragon Institute of MGH, MIT and Harvard,; Perelman School of Medicine, University of Pennsylvania; University of Pennsylvania; Ragon Institute of MGH, MIT and Harvard; University of Pennsylvania; University of Pennsylvania; University of Pennsylvania; University of Pennsylvania; University of Pennsylvania; Hospital of the University of Pennsylvania; University of Pennsylvania; Accelevir Diagnostics; University of Pennsylvania Perelman School of Medicine; University of Pennsylvania; University of Pennsylvania; University of Pennsylvania; University of Pennsylvania; University of Pennsylvania; Perelman School of Medicine University of Pennsylvania; University of Pennsylvania; University of Pennsylvania; University of Pennsylvania; University of Pennsylvania; Ragon Institute of MGH, MIT and Harvard; Ragon Institute of MGH, MIT and Harvard

## Abstract

We conducted a first-in-human evaluation of HIV-specific, HIV-resistant chimeric antigen receptor (CAR) T cells (CARTs) to determine their capacity to modulate viral rebound dynamics following analytic treatment interruption (ATI). Among ten treated individuals who received engineered T cells, one individual did not rebound during the ATI and had a remarkable expansion of their CARTs 43 days after the start of the ATI, suggesting that the CARTs recognized rebounding HIV and prevented its spread. Post-rebound virologic control was observed in 1 participant for 90 weeks and another 5 participants had remarkable reductions in their viral load after viral peak was reached, maintaining viral loads below their set point. CART infusion was associated with broad reinvigoration of endogenous HIV-specific CD8 T cell responses, and immune escape to one of these responses was documented. Together, these findings show that HIV-specific CARTs can be an integral component in HIV Cure strategies.

## INTRODUCTION

Contemporary antiretroviral therapy (ART) durably suppresses plasma viremia, but it does not eradicate long-lived, latently infected cells. Viral rebound is nearly universal when ART is interrupted, necessitating lifetime treatment for people with HIV (PWH). Immune-based therapies are being tested as strategies to maintain HIV suppression without continuous ART by augmenting antiviral mechanisms that are insufficiently effective during natural infection. These approaches include passive immunotherapy with broadly neutralizing antibodies (bNAbs)^1–3^, therapeutic vaccination^4,5^, immune checkpoint modulation^6,7^, and adoptive or engineered cellular therapies^8–11^. bNAbs have advanced furthest clinically because they combine potent neutralization of cell-free virus with Fc-mediated clearance of infected cells and may also enhance endogenous antiviral immunity. Nevertheless, ART-free suppression after bNAb administration remains inconsistent, and recent studies suggest that rapid HIV-specific T-cell responses may be a key determinant of durable post-treatment control^12,13^.

Engineered T cells are an attractive candidate to supply this rapidly responding, durable T cell response to individuals lacking this natural T cell response because they can be endowed with predefined antigen specificity, resistance to infection, engage and re-invigorate other arms of the immune system, and can be engineered for long-term persistence^14–16^. Early clinical experience with HIV-specific CARTs expressed a CD4ζ receptor that uses the extracellular domains of CD4 to bind to HIV Env linked to the CD3 ζ chain to target HIV infected cells^17^. Infusion of CD4ζ-modified T cells into viremic individuals was safe, demonstrated trafficking to gut-associated lymphoid tissue, and produced a modest but transient reduction in rectal tissue-associated HIV RNA, without consistent effects on plasma viremia or evidence of post-rebound control^18–20^. Long-term follow-up across three CD4ζ trials showed that these CAR T cells persisted for more than a decade at stable frequencies, retained functional signaling capacity, and did not exhibit integration-related clonal expansion or transformation, establishing a strong safety and persistence benchmark for T cell gene therapy in HIV^21^. However, because CD4ζ CARs render the engineered cells susceptible to HIV infection and most participants remained on ART without structured interruption, these studies, due to the time in which they were performed, provided limited insight into whether HIV-specific CARTs can actively control viral rebound.

More recent CAR designs have incorporated HIV-specific bNAb-derived antigen-binding domains to harness the breadth and potency of contemporary antibodies. A recent phase I study evaluated a third-generation CAR based on a VRC01 bNAb single-chain variable fragment in individuals on suppressive ART who then underwent ATI (NCT03240328). Administration of a single dose of these CARTs was well tolerated and led to significant reductions in cell-associated HIV RNA and intact proviral DNA, suggesting on-target pressure on the reservoir^8^. Nonetheless, among participants who discontinued ART, plasma viremia rebounded in all cases with a median time to rebound of just over five weeks, and sequencing demonstrated selection of escape variants rather than sustained post-rebound control of replication. Thus, while bNAb-based HIV CARs have established clinical feasibility and reservoir-directed activity, current designs have not yet achieved durable functional cure or consistent control of viremia following ART interruption.

Building on the safety, difficulty of HIV escape, and persistence established by CD4ζ CARs, we hypothesized that an improved CD4 CAR design that incorporated lessons learned from successful CD19-specific CARTs^22,23^ coupled with rendering these T cells HIV resistant by editing their CCR5 gene^24,25^ could result in HIV-specific, HIV-resistant CAR T cells capable of mediating control of viral replication even after systemic rebound during ATI. In this pilot clinical study, we evaluated autologous T cells engineered to express an HIV-specific CAR that used CD4 to redirect T cells to HIV envelope expressing cells and to resist HIV infection through CCR5 gene editing. The product was administered to PWH on suppressive ART, who subsequently underwent an ATI to assess safety, persistence, and antiviral activity *in vivo*. This clinical experiment tested whether HIV-resistant CARTs can persist and function during viral rebound and whether they can alter the kinetics or magnitude of HIV replication after ART withdrawal.

## RESULTS

### Study participants, T cell manufacturing, and safety

Ten participants (10 men; median age 46, range 21–65) were enrolled, all with well-controlled HIV on ART (see CONSORT flow diagram, **Fig S1, Table S1**). Baseline median CD4^+^ T-cell count was 746 cells per μL (range, 508–1,418). ART regimens included an integrase inhibitor (9/10), a boosted protease inhibitor (1/10), and an NRTI (10/10) (**Table S1**). All participants were determined to have initiated ART during chronic HIV infection based on CD4 T cell count at treatment initiation, detailed clinical history, and documented duration of infection (**Table S1**).

**Figure 1:**
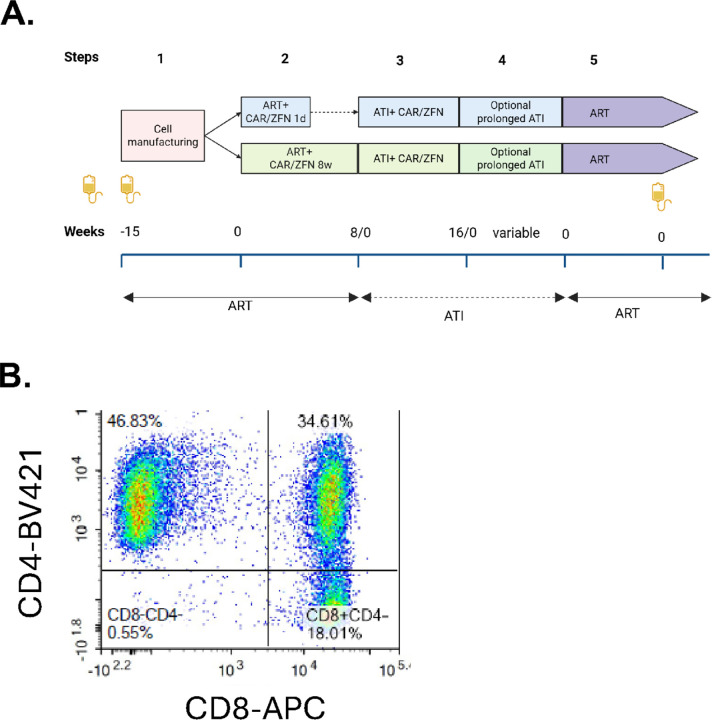
Study participants, T cell manufacturing, and safety: A.) Schematic timeline of study steps and sample collection for NCT03617198. The clinical trial was divided into five steps. Leukapheresis to obtain cells for manufacturing was followed by a second leukapheresis Participants entered step 2 on the day of CCR5-edited CD4BBζ CARTs infusion (day 0), where cells were permitted to engraft for either 1 day (Cohort 1) or greater than 8 weeks (Cohort 2) before initiating a 16-week analytical treatment interruption as step 3. At the conclusion of treatment interruption, participants progressed to step 4 to allow for safety monitoring until HIV RNA levels fell below the limit of quantification or if their viral load remained below 1000 copies/ml, the participants could opt to continue their ATI. In step 5, participants had a final leukapheresis 6 months after ART re-initiation. Yellow blood bag symbol represents when apheresis material was collected. (B) Surface expression of CD4 and CD8 measured by flow cytometry after clinical manufacturing of 10 billion T cells that were stained prior to being infused into participant 01.

The study proceeded in five steps (Fig. 1A). In Step 1, participants underwent leukapheresis in order to manufacture their autologous engineered T cells. Here, isolated T cells were electroporated with CCR5-specific ZFNs^24^, activated with CD3/28 coated beads, and then transduced with a lentiviral vector encoding CAR that used the CD4 extracellular domain as the HIV Env binding entity coupled to the 4–1BB and CD3ζ signaling domains^22^ (CCR5-edited CD4BBζ CARTs), and cultured for at least 9 days (Fig. 1B).

A second leukapheresis was collected to enable correlative studies and serve as a backup T cell source in case issues arose during the initial T cell manufacturing. Cell manufacturing was successful for all participants (**Table S1**). In Step 2, the participants received a single infusion of up to 10 billion autologous engineered T cells (1.8 ×10^9^-1 ×10^10^) that contained between 1.13 and 3.85 billion CCR5-edited CD4BBζ CARTs. Of note, three participants (01, 02, and 08) were heterozygous for Δ32 allele which increased the likelihood that their CCR5-edited cells would be fully HIV resistant. Step 3 consisted of a planned 16-week analytical treatment interruption (ATI), with protocol-defined safety triggers for ART reinitiation. Participants were randomly assigned to be in one of two cohorts: in cohort 1 the ATI was initiated one day after T cell infusion; in cohort 2 the ATI was initiated at least 8 weeks after the T cell infusion. This design was undertaken to assess a possible role for post-infusion maturation and/or expansion of CART cells in viral control In Step 4, participants in consultation with their physician could opt to extend their ATI contingent upon stable CD4 T cell counts and viral load remaining below 1000 copies/ml, and in Step 5 ART was resumed and a final leukapheresis was performed at least 6 months after viral control was re-established so that durable changes to HIV-specific immune response and the HIV reservoir could be studied. The infusions were well tolerated, with no serious adverse events. No cytokine release syndrome (CRS), immune effector cell–associated neurotoxicity syndrome (ICANS), or other clinically significant neurologic toxicity was observed. Overall, 34 adverse events (25 Grade 1, 9 Grade 2) were reported; 30 were considered unrelated and 4 were related in attribution to the infusion of CCR5 edited CD4BBz CARTs (**Table S2 and S3**).

### Viral rebound and post-rebound control during analytical treatment interruption

**Figure 2:**
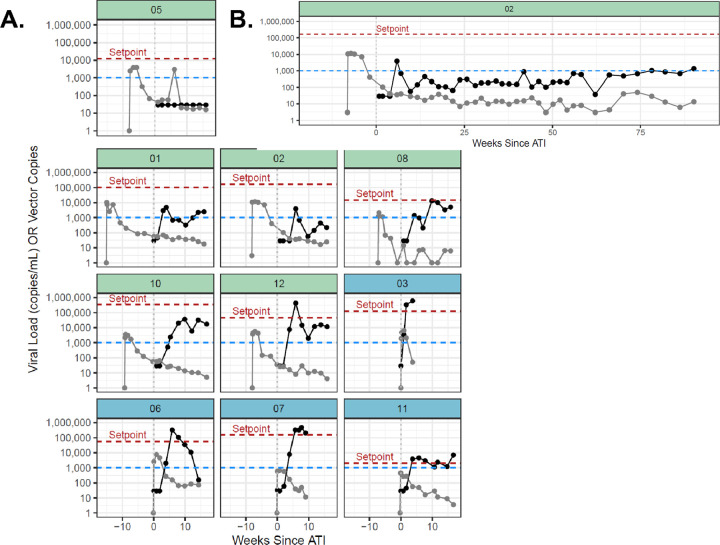
Infusion of CCR5-edited CD4BBζ CARTs results in a lack of viral rebound in one participant and post rebound control of HIV in a subset of participants. A) Viral load (black line) and vector copies per million PBMCs (grey line) are shown for each participant during the ATI. Participants in Cohort 1 have a blue heading and those in Cohort 2 have a green heading. B) Extended ATI for participant 02.

During the ATI, viral rebound occurred in 9 of 10 participants within 1–6 weeks, similar to that reported in historical ATI cohorts^26^ (Fig. 2A). One participant (05) showed no detectable viral rebound 16 weeks following ATI. Unfortunately, this individual opted not to extend ATI beyond 16 weeks, and thus the durability of this control is unknown. During the ATI, antiretroviral drug concentrations in serum were at or below the level of detection, supporting adherence to treatment interruption (**Table S4**). Consistent with a role of CCR5-edited CD4BBz CARTs in preventing viral rebound was a spike in the number of transduced T cells in the peripheral blood that occurred 43 days after ATI initiation (Fig. 2A), possibly reflecting CCR5-edited CD4BBz CARTs responding to and effectively controlling viral rebound.

Among the remaining participants, in 6 of 9 (participants 01, 02, 06, 08, 10, and 12) viral load dropped after rebound and stayed below the pre-ART set point until the end of the ATI, suggesting partial post-rebound control of HIV replication. Interestingly, in all individuals who started their ATI one day after infusion of CART cells (Cohort 1- participants 03, 06, 07 and 11) viral rebound rapidly reached the pre-ART viral set point by 4–5 weeks. However, participant 06 showed an impressive post-rebound decline where 325,246 viral copies were detected at week 6 and 105 viral copies per ml at week 16, while the other individuals in this cohort maintained viral loads at or above set point until ART was restarted. Of note, participant 06 agreed to end his ATI but did not restart ART. He rebounded again and maintained his previous set point for 2 months before restarting ART again (**Fig. S2**). In contrast, all individuals who received their CCR5-edited CD4BBζ CARTs at least 2 months prior to the start of ATI showed some form of immune control and only one participant’s (08) viral load returned to its setpoint. This finding suggests that allowing a longer interval between CART infusion and ATI may favor engraftment and/or functional maturation of the engineered cells before viral challenge.

CD19 and BCMA-specific CARTs expand once infused into people in response to recognizing their cognate antigen^27–29^. We did not observe this type of expansion when HIV-specific CARTs were infused into PWH in 9 of the 10 individuals. In fact, in most cases, the number of HIV CARTs in the peripheral blood rapidly dropped over time (Fig. 2A). Interestingly, while low, the HIV-specific CARTs did persist over time at levels in the same range to those who durably control cancer with CD19-CARTs^27–29^. Surprisingly, we did not see expansion of HIV-specific CARTs even in individuals who rebounded quickly and achieved high viral loads, suggesting that relationship between HIV CART expansion differs from the relationship between tumor and tumor-specific CARTs. One of our participants (02) opted to extend ATI into Step 5 of the protocol, having met the criteria of viral load being at or below 1000 copies/ml. Here, we were able to observe durable post rebound control as this individual maintained a viral load of under 1000 copies/ml for 90 weeks (Fig. 2B). Although this participant never met the protocol-defined criteria to re-start ART, resumption of antiretroviral treatment was eventually made at the 90-week mark upon advice of the treating physician. This prolonged period of low-level viremia provides evidence that post-rebound control can be sustained in selected individuals after CCR5-edited CD4BBζ CAR T-cell infusion, despite high-level pre-ART setpoint viremia.

### No changes in HIV reservoir levels after infusion of CCR5-edited CD4BBζ CART infusion

**Figure 3:**
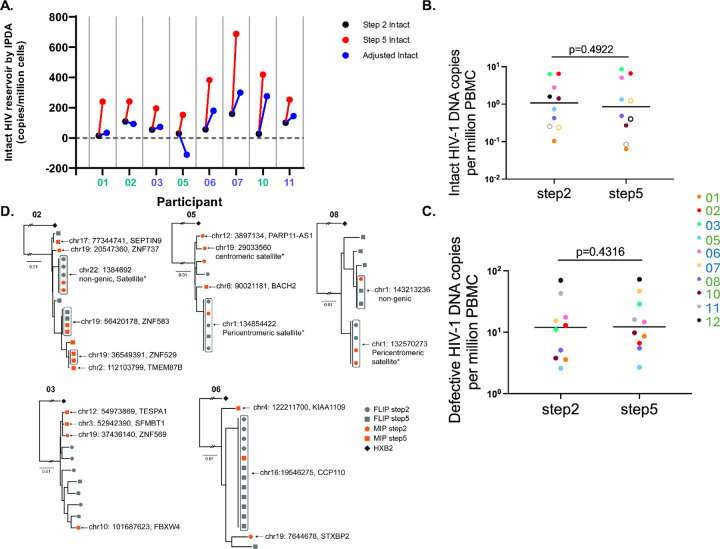
No changes in HIV reservoir levels after infusion of CCR5-edited CD4BBζ CARTs. A) IPDA and the number of vector copies per 1 million CD4 T cells were measured at Step 2 and Step 5 for each participant. Adjusted IPDA at Step 5 reflects subtracting vector copies from intact viral copies. B,C) Frequencies of intact (B) and defective (C) proviruses in individuals at step 2 treatment and step 5. Open circles represent data at limit of detection. Horizontal bars indicate the median. Significance was tested using Wilcoxon matched-pair signed rank tests. D) Linear maximum likelihood phylogenetic trees reflecting intact proviruses isolated from indicated study participants by FLIP-seq or MIP-seq at step 2 or step 5. Chromosomal integration site coordinates (aligned to the HG38 human reference chromosome); integration sites that did not map the HG38 genome were aligned to the human T2T reference genome and are highlighted by *).

We next assessed whether CCR5-edited CD4BBζ CARTs could alter the quantity or composition of the latent persistent HIV reservoir. We first used Intact Proviral DNA Assay (IPDA) to quantify the reservoir^30^. Measurements were obtained from all participants except 08 and 12 whose Ψ sequence was not detected by IPDA primers. Initial IPDA analysis showed an apparent increase in intact DNA proviral copies at the end of the study (Fig. 3A). Because the CD4BBζ CAR was delivered using a third-generation lentiviral vector that contained HIV-derived sequences that are detectable by IPDA primers, we hypothesized that CARTs were contributing to the apparent increase in IPDA-detected intact proviruses. To test this, we quantitated the number of vector copies using primers specific to WPRE sequence located within the lentiviral vector and observed there was a strong correlation between the increase in intact reservoir detected by the IPDA and the number of WPRE sequences. After subtracting the number of WPRE sequences from the intact HIV copies as we previously described^31^, we did not see a difference in reservoir levels after the infusion of HIV-resistant, HIV-specific CARTs (p=.08) though there was still a trend for higher levels after infusion, which may reflect inaccuracies in our subtraction adjustment as participant 05 had a negative intact viral reservoir. Together, these data indicate that CCR5-edited CD4BBζ CAR T-cell infusion did not produce a measurable reduction in the intact HIV reservoir by IPDA, and that unadjusted IPDA values after lentiviral-vector–modified cell infusion require cautious interpretation.

### No consistent change in proviral composition or integration-site distribution after CART infusion

To further evaluate HIV reservoir dynamics during treatment with CCR5-edited CD4BBz CARTs, we performed near–full-length single-genome proviral sequencing (FLIP-seq) and matched integration site and proviral sequencing (MIP-seq) on peripheral blood mononuclear cells (PBMCs) collected before and after CART infusion. These complementary approaches enabled quantification of intact and defective proviruses and precise mapping of proviral integration sites relative to genic and non-genic regions of the human genome. In total, we detected 1294 individual proviral genomes from 119.5 million cells collected before and after CART infusion across 10 participants, of which 74 were identified as genome-intact. The frequency of intact and defective HIV-1 proviruses in PBMCs showed no consistent or statistically significant change following CART infusion, as assessed by FLIP-seq (Fig 3B,C). In several participants, the proportion of clonally expanded intact proviruses relative to the total intact reservoir changed following CART infusion. Phylogenetic analysis of intact proviral sequences demonstrated persistence of expanded clones across time points, consistent with long-lived infected cell populations (Fig 3D). However, shifts in the relative contribution of individual clones suggested differential survival of infected cells rather than uniform clearance, consistent with previous work^32,33^. It is unclear whether the infusion of CCR5-edited CD4BBz CARTs is responsible for these shifts. We also examined where within the genome the reservoir was located. MIP-seq showed no consistent change in the genomic distribution of proviral integration sites after CAR T-cell infusion, including the proportion of integrations in genic versus non-genic regions and other measured genomic features (**Supplemental Table 5**). Of note, in two study participants, 05 (non-rebounder) and 02 (post-rebound controller) had an enrichment of intact proviruses integrated in heterochromatin regions, including centromeric satellite DNA and ZNF genes; moreover, in study participant 08, viral control below a plasma viral load of 10,000 copies/ml during ATI was also associated with an integration site profile of intact proviruses biased towards heterochromatin regions (Figure 3C). In contrast, integration sites of intact proviruses in study persons 03 and 06 were exclusively in genic regions. Together, these data indicate that CCR5-edited CD4BBζ CART infusion did not produce a measurable change in reservoir size, the clonal composition of the viral reservoir cell pool composition, or integration site distribution of intact proviruses in peripheral blood during the period studied. However, individuals whose intact reservoir is enriched in non-genic regions of the genome may be more likely to control HIV replication after the introduction of HIV-specific CARTs.

### HIV-specific CARTs re-invigorate natural HIV-specific CD8 T cells forcing immune escape.

**Figure 4:**
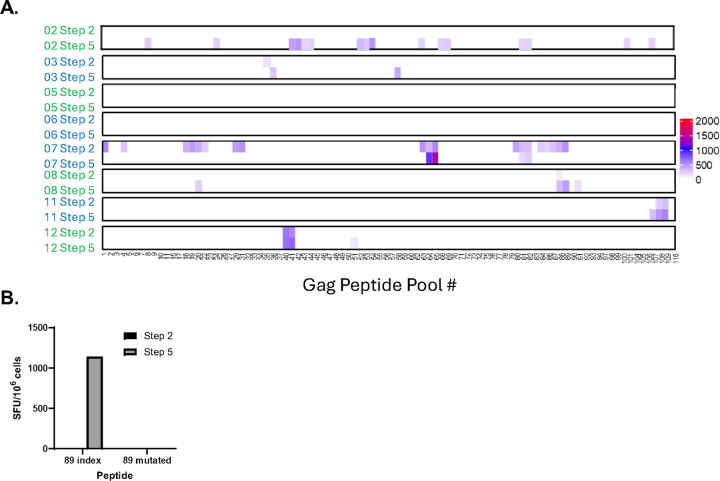
HIV-specific CARTs re-invigorate natural HIV-specific CD8 T cells leading to immune escape: A) Heatmap showing the T cell response against individual peptides from HIV GAG protein, measured as the production of IFNγ by ELiSPOT. Numbers shown were background subtracted and represent the number of IFNγ+ spots per million cells. Apheresis 2 (PRE) and Apheresis 5 (POST) responses are shown. Positive responses were identified before background subtraction, as those equal or above 100 spots forming cells per million cells and 4X above background. Participants 001 and 010 T cell responses were not evaluated against individual peptides because they did not show T cell responses above threshold against GAG peptides pools. B) Intact viral sequences at Step 2 and Step 5 were compared and a change in peptide VGGPGHKARVLAEAM to VGGPQHKARVLAEAMwas detected. Immune recognition of these peptides was detected by ELiSPOT as above.

In addition to directly targeting HIV infected T cells, HIV-specific CD4 T cells and CARTs can restore HIV-specific help and promote natural immune responses to HIV^34^. We next assessed whether CCR5-edited CD4BBζ CAR T-cell infusion was associated with changes in endogenous HIV-specific cellular immunity during ATI. We measured both the breadth and magnitude of the HIV-specific CD8 T cell response to overlapping consensus B gag peptides. While there are some modest changes in the Gag-specific CD8 T cell response before and after the infusion of CCR5-edited CD4BBζ CARTs in individuals in Cohort 1, two of the individuals in Cohort 2 (02 and 08) had remarkable improvements in both the breadth and magnitude of the Gag-specific CD8 T cell response (Fig 4A). Interestingly, participant 05 had no change in their very modest immune response, likely due a lack of viral rebound. The largest increase in Gag-specific CD8 T cell reactivity occurred in participant 02, who maintained plasma HIV RNA below 1,000 copies per mL during extended ATI. Participants who rebounded with very high loads did not have dramatic changes, suggesting low but not uncontrolled viral replication is required for CCR5-edited CD4BBζ CARTs to re-invigorate the natural HIV-specific CD8 T cell response.

To determine whether enhanced CD8 T cell responses exerted detectable immune pressure on the reservoir, we compared intact proviral sequences before and after CCR5-edited CD4BBζ CAR T-cell infusion By examining the sequences of intact viruses before and after CCR5-edited CD4BBζ CART infusion, we observed that all 5 intact viruses sequences detected in participant 08 had a Glycine in position 359 and all 5 intact virus sequences obtained at the end of study had glutamine at the same position. Using index peptide 89, we constructed a mutated version that contained a glutamine residue instead of glycine and tested whether this change resulted in immune escape. Indeed, whereas we observed a robust response to the index peptide using PBMCs isolated at Step 5, we saw no reactivity to the mutated peptide (Fig 4B). This finding strongly suggests that the immune pressure was responsible for this mutation, highlighting the role HIV-specific CARTs can play in promoting HIV cure by enhancing the endogenous HIV-specific immune response.

## DISCUSSION

In this first-in-human study, we evaluated autologous HIV-specific CARTs engineered to target HIV envelope via CD4 binding made resistant to HIV infection through CCR5 editing. Treatment was well tolerated, with no cytokine release syndrome, immune effector cell-associated neurotoxicity syndrome, or other serious treatment-related adverse events. Although most participants experienced viral rebound during ATI, several exhibited evidence of post-rebound virologic control, including one participant (02) who maintained plasma HIV RNA below 1,000 copies mL^−^^1^ for more than 90 weeks without ART and another (06) who following rebound exhibited a rapid decline in viral load during the planned 16-week ATI. Moreover, in one participant (05), no rebound occurred during the ATI. These findings provide clinical evidence that HIV-specific, HIV-resistant CARTs can contribute to control of HIV replication following ART withdrawal.

This study was designed to address two limitations of earlier CD4ζ-based CART approaches: 1) susceptibility of engineered cells to HIV infection and 2) limited evaluation during a structured ART interruption. Early CD4ζ CART studies established the safety and long-term persistence of HIV-directed CARTs^18–20^, but their antiviral activity was modest, and the engineered cells remained vulnerable to HIV infection. We therefore combined a second-generation CD4-based CAR incorporating 4-1BB costimulation domain with CCR5 gene disruption, building on advances in CART engineering and previous clinical studies demonstrating the feasibility of CCR5 editing in humans^9,25^

The virologic outcomes observed during ATI provide the strongest evidence to date of antiviral activity of HIV-specific CARTs. Although rebound occurred in most participants within the expected timeframe^35^, several individuals exhibited reductions in plasma HIV RNA after rebound and maintained levels below their historical pre-ART set points. The prolonged control observed in participant 02 and the absence of rebound in participant 05 suggest that HIV-specific CAR T cells can alter post-treatment viral dynamics in at least a subset of individuals. Given the small sample size and lack of a control arm, these findings should be interpreted cautiously; however, the observed patterns differ from those typically reported following ATI alone^35^ and support further evaluation in larger studies. An intriguing observation was that participants who delayed ATI for at least 2 months after CART infusion appeared more likely to achieve virologic control than those who initiated ATI immediately after treatment. This finding raises the possibility that CARTs require time to engraft, distribute to relevant anatomical sites, and establish durable memory populations before viral challenge. Consistent with this interpretation, participant 05 demonstrated a marked increase in circulating CAR T cells more than 40 days after ATI initiation despite the absence of detectable viremia. Although speculative, this expansion may reflect recognition and containment of low-level viral recrudescence in tissues before systemic rebound became detectable. Future studies should evaluate ATI timing as a potentially important determinant of efficacy.

Unlike CD19- and BCMA-directed CAR T-cell therapies^27–29^, HIV-specific CARTs did not undergo substantial peripheral expansion after infusion, even in participants with high-level viremia. Similar observations have been reported in non-human primate models of HIV-specific CAR T-cell therapy^36^. This distinction likely reflects fundamental biological differences between cancer and HIV infection. Whereas malignant and healthy B cells provide abundant and continuously expressed antigen, HIV-infected cells are rare, anatomically dispersed, and often express viral proteins only transiently^37^. Consequently, successful HIV CART therapy may depend less on massive systemic expansion and more on persistence within tissue reservoirs where viral reactivation occurs. The degree of HIV resistance achieved in the infused product may represent an important determinant of efficacy. Several participants (01,02, and 08) who demonstrated evidence of post-rebound control were heterozygous for the CCR5Δ32 allele, potentially increasing the frequency of fully HIV-resistant CARTs. Because current GMP-compatible manufacturing methods generate only modest levels of biallelic CCR5 disruption, most infused CARTs likely remained susceptible to infection. Although these observations are hypothesis-generating, they suggest that improving the efficiency of HIV-resistance engineering may enhance CART survival, expansion, and antiviral function during viral rebound.

A notable mechanistic finding was the enhancement of endogenous HIV-specific CD8 T cell responses following CART infusion. Recent studies of post-treatment controllers and antibody-mediated remission strategies have identified rapid HIV-specific T-cell responses as a key correlate of sustained viral control^12,13^. Participants with the greatest increases in HIV-specific CD8 T cell responses generally exhibited the strongest virologic control. In participant 08, emergence of a Gag escape mutation that abolished T cell recognition provided direct evidence of immune pressure on replicating virus. These findings suggest that HIV-specific CARTs may function through both direct elimination of infected cells and indirect amplification of endogenous antiviral immunity, consistent with preclinical observations that CD4^+^ CARTs can provide helper functions to endogenous immune responses^9,34^.

Despite evidence of antiviral activity, we did not detect consistent changes in reservoir size, proviral composition, or integration-site distribution. This was not unexpected given the stability of the latent HIV reservoir and the relatively short observation period^38^. The absence of measurable reservoir depletion suggests that the virologic control observed in selected participants was driven primarily by immune-mediated containment of viral replication rather than substantial elimination of infected cells. These findings support a growing view that durable ART-free remission may be achievable without complete eradication of the viral reservoir.

Several limitations should be acknowledged. This was a small pilot study without a control arm and included only male participants. Participant-specific factors, including prior receipt of CCR5-edited CD4 T cells in participant 02 (participant 205^25^ and participant 301^9^), may also have influenced outcomes. Finally, most analyses were performed in peripheral blood and may not adequately reflect CART persistence or activity within lymphoid and mucosal tissues, where HIV persists. Together, these results demonstrate that HIV-specific, HIV-resistant CARTs can be safely administered to PWH and can exert measurable antiviral activity *in vivo*. The data support a model in which these cells contribute to viral control through both direct targeting of infected cells and enhancement of endogenous HIV-specific immunity. Future studies should focus on increasing the proportion of HIV-resistant CAR T cells, defining and perhaps promoting their tissue distribution and persistence, optimizing ATI timing, and evaluating combinations with broadly neutralizing antibodies, therapeutic vaccines, and latency-targeting interventions. As HIV cure strategies continue to evolve, HIV-specific CARTs may provide a durable cellular component capable of recognizing rebounding virus and maintaining long-term immune surveillance in the absence of ART.

## MATERIALS AND METHODS

### Clinical Trial:

The study was conducted under IRB approved protocol 831464 and registered under NCT03617198. The trial opened to enrollment on July 31, 2019 with a clinical data cutoff date of November 15, 2024.

### T cell manufacturing:

CCR5-specific ZFN sequences using previously published sequences^24,39,40^, were codon optimized, and placed in pIDTSmart vector (Integrated DNA Technologies). GMP grade RNA was generated as previously described^9^. Autologous peripheral blood lymphocytes were obtained via leukapheresis collection. Leukapheresis products from the participants were enriched for T cells by depletion of monocytes via counterflow centrifugal elutriation. The resulting enriched T cells were electroporated with ZFN mRNAs in a closed system using the MaxCyte GT Flow Transfection System. Cells were next incubated at 30°C for 2 days prior to activation with anti-CD3/anti-CD28 mAb–coated paramagnetic beads (Dynabeads CD3/CD28 CTS, 43200D, Thermo Fisher Scientific,). The cells were then transduced with GMP grade lentiviral vector encoding CD4BBz CAR^22^ the following day and cultured in a closed system at 37°C with complete X-VIVO 15 media (Lonza) supplemented with IL7 and IL15. Two days after transduction, media was also supplemented with the antiretroviral drugs Retrovir and Norvir (Abbott Laboratories). T cell expansion continued after transfer to a WAVE Bioreactor (General Electric) for additional expansion under perfusion conditions up to 11 days after T cells activation. At the end of the culture period, cells were depleted of magnetic beads, washed, and formulated in infusible cryopreservation media. Prior to release, cells were tested for sentinel viability (≥70%); residual bead numbers (≤100 beads per 3 × 10^6^ cells); bacterial and fungal contamination (no growth by day 7) and mycoplasma contamination (negative); HIVGag provirus copies (post-expansion numbers not statistically greater than pre-expansion numbers); percentage of CD3/CD45 cells greater than or equal to 80%; endotoxin levels of 3.5 EU/mL or less; CCR5 gene disruption (detectable); and at least 20% of CD8 T cells expressing CD4. Flow cytometry was performed as previously described^31^ and used the following antibodies: CD3 PE, Clone SK7 (BD Biosciences, Cat# 347347), CD4 BV421, Clone OTK4 (BioLegend, Cat# 317434), CD8 APC, Clone SK1 (BD Biosciences, Cat# 340584), CD45 BV605, Clone HI30 (BioLegend, Cat# 304042). Serum ART levels were measured by UNC CFAR Clinical Pharmacology and Analytical Chemistry Core.

### HIV IPDA and lentiviral vector quantification by WPRE:

The HIV-1 subtype B IPDA^®^ was performed on CD4^+^ T cells isolated from cryopreserved PBMCs by Accelevir Diagnostics under company standard operating procedures. An in-depth description of the assay design and performance benchmarks in PWH on ART have been previously described^30,41^. To quantify persistent lentiviral vector integrants, we performed a ddPCR assay consisting of two independent reactions, one targeting the WPRE enhancer sequence found in the vector and the other targeting the human RPP30 copy reference gene. This assay was performed the DNA isolated from peripheral CD4^+^ T cells which remained after IPDA^®^ testing, and where possible DNA input was matched between assays. WPRE quantities were reported as copies per million input CD4^+^ T cell genomes. The HIV-1 subtype B IPDA^®^ detects two HIV-1 proviral DNA regions (psi element and Rev response element, or RRE) which are also found in the lentiviral CART vector used in this study. As such, persistent vector integrants can also contribute to the signal detected by IPDA^®^, complicating the interpretation of intact proviral quantification data. Under the assumption that each WPRE copy detected here came from an intact CART vector integrant that also contained a single copy of the HIV-1 psi element and RRE, we subtracted the WPRE copy number from the final IPDA^®^ reported quantity of intact HIV-1 proviruses to yield a corrected measurement of HIV-1 reservoir size.

### Near full-length individual HIV proviral genome sequencing (FLIP-seq)

FLIP-Seq was conducted as previously described. Briefly, genomic DNA diluted to single-genome levels was subjected to HIV-1 near full-genome amplification using Invitrogen Platinum Taq with nested primers described previously^42^, except for replacing the second-round nested reverse primer with the following primer sequence: 5’-AGTACAGGCAAAAAGCAGCTGCTTA-3’. MIP-Seq was performed on genomic DNA subjected to whole-genome amplification (WGA) using phi29 DNA polymerase (#150345; Qiagen REPLI-g Single Cell Kit) according to the manufacturer’s instructions, as previously described^43^. Following WGA, amplified DNA from each well was split and independently used for near full-length viral sequencing and integration site analysis, as described below. For both FLIP-Seq and MIP-Seq workflows, PCR products were visualized by agarose gel electrophoresis. Near full-length amplicons were sequenced on an Illumina MiSeq platform (MGH DNA Core Facility). Raw reads were de novo assembled using UltraCycler v1.0 and aligned to the HIV-1 HXB2 reference genome to identify large internal deletions (<8,000 bp aligned to HXB2), frameshifts or out-of-frame indels, premature stop codons, internal inversions, and packaging signal defects (defined as ≥15 bp insertions/deletions relative to HXB2). Sequence classification was performed using an automated in-house Python pipeline (https://github.com/BWH-Lichterfeld-Lab/Intactness-Pipeline). APOBEC3G/3F-associated hypermutations were assessed using the Los Alamos National Laboratory (LANL) Hypermut 3.0 tool. Sequences lacking any of the defects listed above were classified as genome-intact. Multiple sequence alignments were generated using MAFFT in Geneious Prime (https://www.geneious.com/) and visualized with Highlighter plots (https://www.hiv.lanl.gov). Viral sequences were defined as clonal when they exhibited identical sequences; single-nucleotide differences within primer binding regions were excluded from clonality assessment. Clade assignment of intact HIV-1 proviral sequences was performed using the LANL Recombinant Identification Program.

### Integration site analysis

Integration sites associated with each viral sequence were obtained using integration site loop amplification (ISLA), as previously described^44^, using DNA derived from whole-genome amplification as template. Biocomputational identification of integration sites was performed as previously described^43,45^. Chromosomal coordinates and gene annotations were assigned using Ensembl (https://www.ensembl.org), the UCSC Genome Browser (https://genome.ucsc.edu), and GENCODE (v39; https://www.gencodegenes.org). Integration events located within repetitive genomic regions were further characterized using RepeatMasker (https://www.repeatmasker.org).

### Measurement of HIV-gag specific CD8 T cell responses:

HIV-1 Clade B gag peptide array, consisting of 123 peptides of 15-mers with 11 amino acid overlap, was provided by the NIH HIV Reagent Program, Division of AIDS, NIAID, NIH (ARP-8117). Peptides were reconstituted in DMSO at 12.5 mg/ml and peptide pools of 31 peptides were created by combining equal amounts of peptides for the first round of ELISpot assays (mapping pools). Peptides were used at a working dilution of 1 mg/mL. The software program Deconvolute This!^46^ was used to optimize matrix configuration, instruct pooling of peptides, and determine second-round assays based on experimental results. With this software, each peptide was assigned to 4 different peptide pools and 16 mapping pools were created and assigned ID numbers from 1 to 16. Of the complete set of 123 peptides, individual stimulating peptides were identified by the results obtained with the overlapping mapping pool of 31 peptides in the first set of ELISpot assays. Because the mapping pools have been compiled in such a way that every single peptide was contained in exactly 4 pools, any peptide inducing a T cell response would result in 4 pools becoming positive. Therefore, the second round of ELISpot assays against individual peptides were designed by inputting in the software the peptide pool ID where a T cell response above threshold was observed (equal or more than 220 spots forming cells per million PBMC and 4 times above background) in the first round of testing. Peptides with spot values above the minimum threshold for all four pools were chosen to be tested individually in a second round of assays. The threshold to identify T cell responses against individual peptides in the second run of ELISpot assays was established at 100 spots forming cells per million PBMC and 4 times above background. IFNg was measured using an ELISPot Plus kit following the manufacturer’s protocol (Mabtech, Cincinnati OH).

## Supplementary Files

This is a list of supplementary files associated with this preprint. Click to download.

- SupplementalData.pdf
